# Supplementary material for: Saccharomyces boulardii (CNCM I-745) improves intestinal damage in sepsis by remodeling intestinal flora structure
Source: Front Cell Infect Microbiol. 2025 Jul 31;15:1602792. doi: 10.3389/fcimb.2025.1602792 (PMC12350329; doi:10.3389/fcimb.2025.1602792)
Supplement: Supplementary file 1 [file DataSheet1.zip › summary/summary/6_Taxonomy_community/1_Taxonomy_profiling/3_Class/All/Class_abund_top30_sample.heatmap.pdf]

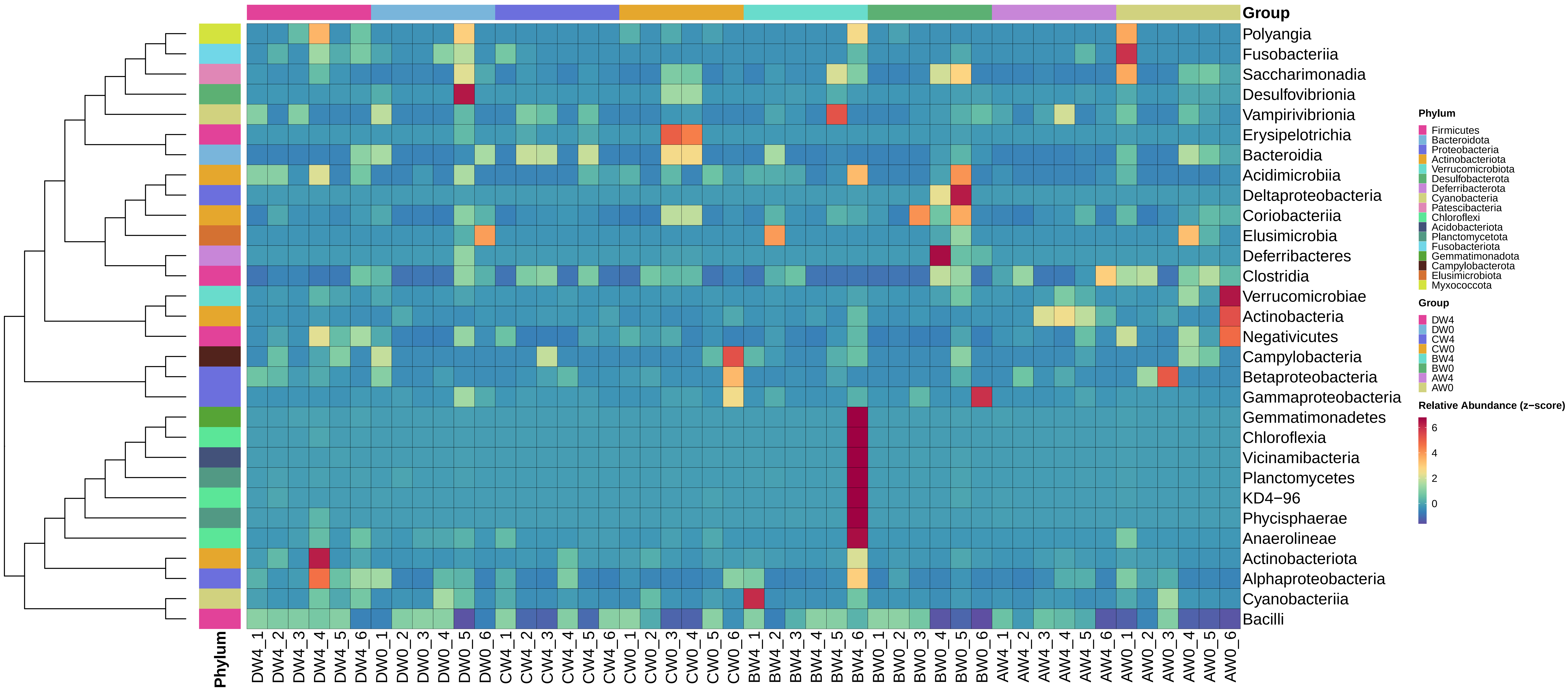
Group
Polyangia
Fusobacteriia
Saccharimonadia
Desulfovibrionia
Vampirivibrionia
Erysipelotrichia
Bacteroidia
Acidimicrobiia
Deltaproteobacteria
Coriobacteriia
Elusimicrobia
Deferribacteres
Clostridia
Verrucomicrobiae
Actinobacteria
Negativicutes
Campylobacteria
Betaproteobacteria
Gammaproteobacteria
Gemmatimonadetes
Chloroflexia
Vicinamibacteria
Planctomycetes
KD4−96
Phycisphaerae
Anaerolineae
Actinobacteriota
Alphaproteobacteria
Cyanobacteriia
Bacilli
Phylum
DW4_1 DW4_2 DW4_3 DW4_4 DW4_5 DW4_6 DW0_1 DW0_2 DW0_3 DW0_4 DW0_5 DW0_6 CW4_1 CW4_2 CW4_3 CW4_4 CW4_5 CW4_6 CW0_1 CW0_2 CW0_3 CW0_4 CW0_5 CW0_6 BW4_1 BW4_2 BW4_3 BW4_4 BW4_5 BW4_6 BW0_1 BW0_2 BW0_3 BW0_4 BW0_5 BW0_6 AW4_1 AW4_2 AW4_3 AW4_4 AW4_5 AW4_6 AW0_1 AW0_2 AW0_3 AW0_4 AW0_5 AW0_6
Phylum
Firmicutes
Bacteroidota
Proteobacteria
Actinobacteriota
Verrucomicrobiota
Desulfobacterota
Deferribacterota
Cyanobacteria
Patescibacteria
Chloroflexi
Acidobacteriota
Planctomycetota
Fusobacteriota
Gemmatimonadota
Campylobacterota
Elusimicrobiota
Myxococcota
Group
DW4
DW0
CW4
CW0
BW4
BW0
AW4
AW0
Relative Abundance (z−score)
6
4
2
0
